# The Cost of Anonymity in the Sharing Economy: Consumers Distrust and Avoid Sellers Without Profile Photos

**DOI:** 10.5334/irsp.991

**Published:** 2025-08-08

**Authors:** Bastian Jaeger, Emir Efendić

**Affiliations:** 1 Tilburg University, NL; 2 Maastricht University, NL

**Keywords:** sharing economy, profile photos, trust, consumer decision-making, privacy

## Abstract

Sharing economy platforms, such as Airbnb, encourage sellers to display profile photos and other personal information to increase consumer trust and engagement. However, research has shown that consumers rely on this information to discriminate against sellers with certain characteristics (e.g., ethnic minorities). Some sharing economy sellers may therefore choose not to display a profile photo because they wish to conceal their appearance or social identity to prevent discrimination or other unfavorable treatment by consumers or because of general privacy concerns. In four preregistered studies with samples from the United States, United Kingdom, and the Netherlands, we examined the consequences of withholding profile profiles. We tested how the presence (vs. absence) of personal photos affects consumer trust and preferences for different sellers. Three experimental studies (total *N* = 380) suggest that consumers distrust and avoid hosts without a profile photo. In Study 4, we analyzed 461 ride-sharing listings and found that drivers with a profile photo charge higher prices for otherwise equivalent rides. In sum, our results suggest that sharing economy sellers face a tradeoff between anonymity and earning opportunities.

## The Cost of Anonymity in the Sharing Economy

### Consumers distrust and avoid sellers without profile photos

Sharing economy platforms enable private citizens to offer various services, such as accommodation (e.g., Airbnb, Couchsurfing) or transportation (e.g., Uber, BlaBlaCar; Einav et al. 2016). Most of these services involve direct contact between potential customers and sellers, but customers may be reluctant to enter the home or car of a complete stranger. Trust, often defined as the ‘intention to accept vulnerability based upon the positive expectations of the intentions or behavior of another’ (Rousseau et al. 1998, p. 395),^1^ is therefore crucial for the functioning of these platforms (Guttentag 2013; Kong et al. 2020; Olivero & Lunt 2004). A key task of the company, which usually exacts a fee on every transaction or operates with paid subscriptions, is to facilitate trust between sellers and consumers by providing them with relevant information (Guttentag 2013). For example, Ert and colleagues’ (2016) general model of consumer decision-making on Airbnb distinguishes between two key factors that influence booking decisions: product attributes, such as the price, size, or location of a listing, and host attributes, such as review scores, photos, and other personal information. These types of information are available on almost all sharing economy platforms. Furthermore, almost all sharing economy platforms feature reputation systems that provide information on how satisfied previous customers were with a certain seller. Sellers are also required, or strongly encouraged, to disclose personal information about themselves, including profile photos, names, and personal descriptions.

Consumers can then rely on information about hosts to form impressions of their trustworthiness and other personal attributes that may be perceived as relevant for informing their booking decisions (Ert, Fleischer & Magen 2016). Evidence for this model was provided by a systematic review that identified more than a dozen factors that have been shown to influence consumer trust in customer-to-customer e-commerce settings (Mayayise 2023). This list included personal information on sellers such as the availability of profile photos and the social identity of the seller. In turn, trustworthiness impressions (and impressions along other relevant dimensions such as attractiveness; Jaeger et al. 2019) have been shown to influence consumer decision-making. A survey study of Airbnb users showed that perceived information quality (e.g., perceiving that Airbnb provides useful information on hosts) was associated with trust, which, in turn, predicted users’ intentions to keep using the platform (Kong et al. 2020). The perceived trustworthiness of specific sellers, based on a profile photo, also predicted decisions in studies that simulated sharing economy interactions, and this was observed when perceived trustworthiness was measured (Teubner et al. 2014) or manipulated (Nancy et al. 2018). Thus, personal information about sellers can improve consumer trust and downstream consequences such as engagement with the platform and positive word-of-mouth (Kong et al. 2020; Mayayise 2023; Teubner et al. 2014).

However, sellers may be reluctant to share personal photos and other information (Dillahunt & Malone 2015; Olivero & Lunt 2004; Ranzini et al. 2017). This motivation may partly be guided by accumulating evidence that suggests that especially the disclosure of profile photos can have negative effects for certain sellers because it enables discrimination (Köbis, Soraperra & Shalvi 2020). In an experimental study, Norwegian participants were less willing to stay with an Airbnb host if the profile photo showed a person from a racial outgroup (e.g., ‘Abdi from Somalia’ vs. ‘Martin from Norway’; Nødtvedt et al. 2021). Analyses of real-world Airbnb listings also suggest that hosts from racial minorities are less popular among potential guests (Edelman & Luca 2014; Jaeger et al. 2019; Jaeger & Sleegers 2023; Marchenko 2019). If guests prefer White hosts, then these hosts should be able to charge higher prices for qualitatively similar listings (Rosen 1974). In other words, if race still predicts the price of listings even after controlling for apartment size and location, review scores, number of reviews, and other factors that may (a) determine consumers’ willingness to pay for a listing and (b) systematically differ between hosts with different racial backgrounds, then this would suggest that price disparities are due to racial preferences of potential customers. In line with this reasoning, Jaeger and Sleegers (2023) found a price disparity of 2.7% when comparing White and non-White hosts in a sample of almost 100,000 Airbnb listings from 14 countries, after controlling for more than a dozen factors that could vary between White and non-White hosts.

Evidence for discrimination has also been found for other sharing economy platforms, such as Uber, Lyft, and BlaBlaCar (Farajallah, Hammond & Pénard, 2016; Ge et al. 2020), and along dimensions other than race, such as attractiveness (Jaeger et al. 2019; Peng et al. 2020) and disability status (Ameri et al. 2020; see also Troncoso & Luo 2023). The fact that these disparities are observed even when review scores, the attractiveness of rentals, and other seemingly relevant factors are held constant in experimental studies (e.g., Nødtvedt et al. 2021), or controlled for in correlational studies (e.g., Jaeger & Sleegers 2023) suggest that at least some consumers avoid sellers from racial minorities *because of their racial background* (what is often referred to as ‘taste-based’ discrimination; Becker 2010), not because they expect that sellers from racial minorities offer a worse product or service (what is often referred to as ‘statistical’ discrimination; Becker 2010). Thus, especially sellers from racial minorities or other groups that are the frequent target of discrimination may be reluctant to display profile photos or other personal information.

### The effect of photo availability on consumer choice and trust

Sellers may choose to withhold personal photos from their profile because of privacy concerns, to reduce the potential for discrimination, or for other reasons (Dillahunt & Malone 2015; Jang 2023; Penman, Vedantam & Nesterak 2016). Yet, increased anonymity, and the removal of profile photos in particular, may also have negative consequences for sellers. People rely on a person’s facial appearance to form judgments about them (Todorov et al. 2015). In the sharing economy, evaluating a sellers’ trustworthiness is of particular importance, and cross-cultural work has shown that trustworthiness is a fundamental dimension on which people evaluate others (Jones et al. 2021). For example, people are perceived as more trustworthy if they look more attractive (Batres & Shiramizu 2022) and if they smile more (Gill et al. 2014). Although face-based trustworthiness impressions show little to no accuracy (Bonnefon, Hopfensitz & De Neys 2017; Jaeger et al. 2022), these impressions are formed quickly and spontaneously (Stewart et al. 2012; Todorov, Pakrashi & Oosterhof 2009).

On most sharing economy platforms, profile photos are featured prominently on sellers’ profiles and previous research confirms that customers rely on profile photos to assess sellers’ trustworthiness (Dai et al. 2018; Ert & Fleischer 2019). Thus, given that trust is crucial and given that trustworthiness judgments are based (at least in part) on a seller’s photo, customers may avoid sellers who do not display a photo. Although we focus on the role of distrust in explaining people’s potential preference for hosts who display personal photos, there are also other mechanisms that could explain such a preference. Faces attract attention and are processed quickly and effortlessly (also see Reber et al. 2004; Freeman & Johnson 2016). It is possible that the ease with which hosts can be judged in the presence of a profile photo may lead to positive affect (Winkielman & Cacioppo 2001), which, in turn, could lead to a more positive assessment of the host.

There is limited evidence on how the presence (vs. absence) of profile photos influences consumer decisions in the sharing economy. An analysis of 45,000 Airbnb hosts in the United States and China showed that hosts without a photo received lower review scores (and this difference was larger in the United States; Jang 2023). However, guests provided review scores after staying with the host. Although some confounds were controlled for (number of bedrooms and bathrooms, property type, and price), it is plausible that the difference in review scores can be explained by other host characteristics rather than the absence of a profile photo per se (e.g., hosts who choose not to post a profile photo might also be less professional or attentive toward guests during their stay).

A study by Teubner and colleagues (2014) tested the causal influence of profile photos more directly. Groups of participants repeatedly engaged in a resource-sharing task. Participants either saw profile photos of the other participants in their group, an avatar that somewhat resembled participants, or a simple silhouette with a letter indicating the gender of the participant. Participants were more likely to share a monetary endowment when they could see profile photos of the other players, and this was partly explained by greater levels of trust. However, the study design was highly abstract and many cues that customers have access to in the sharing economy were not captured in the paradigm. This limitation was somewhat improved in another study in which participants viewed simplified, hypothetical Airbnb listings with varying prices, review scores, and profile photos (Fagerstrøm et al. 2017). When no photo of the host was displayed, participants were less motivated to explore the listing and less likely to rent the apartment. Although their study design more closely captured the decision-making context of a sharing economy platform, Fagerstrøm and colleagues (2017) used images from a face database in their study, with individuals who were instructed to show specific facial expressions, rather than naturalistic photos commonly used on sharing economy platforms. Moreover, participants saw very limited information about the listing and, crucially, no other personal information about the host that is usually visible on Airbnb and other platforms. This may inflate the effect of photo availability on booking preferences because photos were the only piece of information about the host that participants had access to.

## The Present Studies

Here, we investigate how the presence vs. absence of profile photos influences consumer preferences and trust in the sharing economy in four preregistered studies. We test the causal effect of removing profile photos from sharing economy listings in a series of lab experiments in which participants indicate their (hypothetical) willingness to stay with different hosts after viewing their profiles. We extend and improve on previous work (e.g., Fagerstrøm et al. 2017) in several key ways. Crucially, we examine consumer preferences and trust using a more realistic model of the decision-making environment. We use actual profile photos and participants also have access to other information about the host (e.g., their gender, age, and a short ‘about me’ section). We also examine the effects of profile photos in the context of another sharing economy platform that has received less attention in previous studies: Couchsurfing. Couchsurfing connects travelers with hosts who are willing to share their home at no cost and, according to the website, they offer a worldwide network of 12 million users across 200,000 cities (www.couchsurfing.com/).

In Studies 1–3 (*N* = 380), we test whether people are more willing to stay with a Couchsurfing host when the host displays a profile photo with their listing. We also explore the role of trust and whether different sellers face different penalties when choosing to omit a personal photo. Specifically, we test whether the absence of a profile photo has a more negative effect for hosts who might experience a benefit from revealing their appearance (i.e., attractive hosts), compared to hosts who might experience a cost from revealing their appearance (i.e., unattractive hosts). In Study 4, we extend this work to the field by examining whether people’s preference for profile photos (and the resulting greater demand for services by sellers who disclose a personal photo) is reflected in the prices of advertised rides on BlaBlaCar (*n* = 461), a long-distance ride-sharing platform (www.blablacar.com).

### Study 1

**Figure 1:**
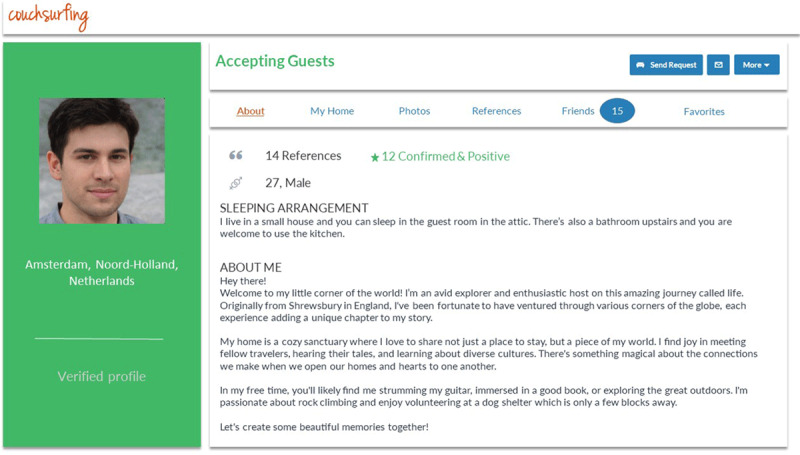
Example of a Couchsurfing listing that participants viewed in Studies 1–3. *Note*. In our studies we used the text and photos from real Couchsurfing profiles. To preserve the anonymity of hosts, this example uses an AI-generated photo and text that was generated by ChatGPT. In one condition, the host’s profile photo was visible. In the other condition, it was replaced with a white silhouette against a gray background.

In Study 1, we examined the effect of profile photos on consumer preferences within the Couchsurfing platform. We created simplified versions of real Couchsurfing profiles (see Figure 1) and asked participants to examine the profiles and indicate their willingness to stay with each host. Each Couchsurfing profile includes a profile photo next to various other information about the host of their home (the sleeping arrangement, hosts’ interests and likes, a review score, etc.). Crucially, we manipulated whether profiles were shown with or without a personal photo of hosts. This allowed us to examine the effect of profile photos on participants’ preferences while keeping all other information about the host and their home constant.

#### Methods

This study was preregistered (see https://osf.io/y4e93/).

**Participants**. The results of an a priori power analysis are reported in the Supplemental Materials. We recruited 95 psychology students at a Dutch university who participated in return for partial course credit. Data from 19 participants (20%) who indicated poor or basic English proficiency were excluded, leaving a final sample of 76 participants (47.37% female; *M*_age_ = 20.82, *SD*_age_ = 1.97).

**Materials**. We created 32 simplified versions of Couchsurfing profiles (see Figure 1). The profiles were based on those of actual hosts from Amsterdam, whose apartments were available for guests on 29 June 2017. Profiles of the first 16 male and 16 female hosts on the website were selected. We created simplified profiles based on the information that is displayed when people first access a host’s profile. This included a host’s profile photo, age, gender, their number of references, a short text about the host (where hosts typically describe themselves or their interests), and the sleeping arrangement (e.g., separate bed in a private room, couch in the living room). The profile photos were similar to the types of photos that are displayed on social media platforms. All photos showed the face (one host wore sunglasses) and parts of the upper body of the host. No other people were displayed in the foreground. Hosts’ age ranged from 19 to 60, but almost all hosts were older than 21 and younger than 40. Names of hosts were omitted. This procedure ensured that participants were exposed to realistic and representative information that they would encounter on the actual website while hosts remained unidentifiable, and participants could scan and respond to multiple listings within the time frame of the study. Next, we created four sets of stimuli. Each set contained all profiles. We varied which random quarter of profiles was displayed without a profile photo.

**Procedure**. Participants were randomly assigned to one of the four sets of profiles. They were asked to imagine they were looking for a place to stay in Amsterdam for the weekend. After reading a short text about the Couchsurfing network, they viewed the 32 profiles in a random order. To measure host preferences, participants were asked to rate their willingness to stay with a given host on a scale from -3 (*I would not consider staying with this person*) to 3 (*I would really like to stay with this person*). We also measured whether participants had ever made use of Couchsurfing and, if not, whether they would consider doing so in the future.

#### Results and discussion

On average, participants’ willingness to stay with the hosts was slightly above the midpoint of our scale (*M* = 0.11, *SD* = 1.72). Participants’ average willingness to stay across all hosts ranged from –1.59 to 2.00 (on our scale that ranged from –3 to 3). The average minimum willingness to stay with specific hosts was –2.67, whereas the average maximum willingness to stay was 2.70. Thus, even though the mean willingness to stay with hosts across all trials and participants was around the midpoint of our scale, there was substantial variation across different hosts and across different participants. Eight participants (10.53%) reported having used Couchsurfing before and 40 participants (52.63%) indicated that they would consider Couchsurfing in the future.

**Figure 2:**
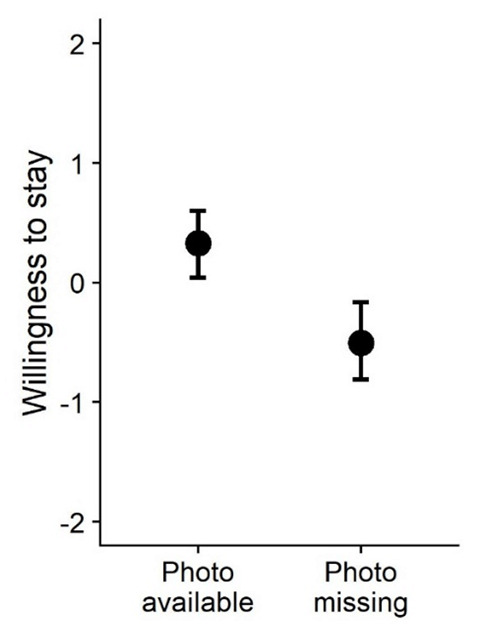
Participants’ willingness to stay with hosts with and without profile photos (Study 1). *Note*. The graph displays predicted means and 95% CIs.

To test how willingness to stay was affected by the presence vs. absence of a profile photo, we estimated a multilevel regression model with random intercepts and random slopes per listing and per participant. Regressing willingness to stay on a dummy variable indicating whether the profile included a photo (0 = photo missing, 1 = photo available) revealed a positive effect *β* = 0.846, *SE* = 0.124, 95% confidence interval (CI) [0.596, 1.092], *t*(50.6) = 6.82, *p* < .001. Thus, holding all other information in a profile constant, participants were more reluctant to stay with hosts without a profile photo (see Figure 2).

### Study 2

In Study 2, we attempted to replicate the results of Study 1 in a larger sample of participants. Crucially, we also investigated the role of trust in explaining consumers’ avoidance of hosts without profile photos. We examined (a) whether participants would show greater distrust toward Couchsurfing hosts when they do not include a personal photo in their profile and (b) whether lower trust can explain why participants are less willing to stay with hosts without profile photos.

#### Methods

This study was preregistered (see https://osf.io/y4e93/).

**Participants**. The results of an a priori power analysis are reported in the Supplemental Materials. We recruited 168 psychology students at a Dutch University who participated in return for partial course credit. Data from two participants (1.79%) who always indicated the same willingness to stay or trust rating and from 12 participants (7.12%) who indicated poor or basic English proficiency were excluded, leaving a final sample of 154 participants (62.29% female; *M*_age_ = 21.93, *SD*_age_ = 4.32). Thus, our sample was slightly smaller than our preregistered sample size.

**Materials and Procedure**. We selected 18 profiles of nine male and nine female hosts that were also used in Study 1. Next, we created three sets of stimuli. Each set contained all profiles. We varied which random third of profiles was displayed without a profile photo. Participants were randomly assigned to one of the three sets and they viewed the profiles in a random order. To measure host preferences, participants were again asked to rate their willingness to stay with a given host on a scale from –3 (*I would not consider staying with this person*) to 3 (*I would really like to stay with this person*). To measure trust in hosts, participants then saw each profile again and they were asked to rate how much they think they can trust the host on a scale from –3 (*I do not trust this person at all*) to 3 (*I trust this person very much*). We also measured whether participants had ever made use of Couchsurfing and, if not, whether they would consider doing so in the future.

#### Results and discussion

Average levels of trust in hosts (*M* = 0.14, *SD* = 1.78) and willingness to stay with hosts (*M* = 0.05, *SD* = 1.86) were slightly above the midpoint of our scale. Participants’ average willingness to stay across all hosts ranged from –2.83 to 1.94 (on our scale that ranged from –3 to 3). The average minimum willingness to stay with specific hosts was –2.52, whereas the average maximum willingness to stay was 2.50. Thus, even though the mean willingness to stay with hosts across all trials and participants was around the midpoint of our scale, there was substantial variation across different hosts and across different participants. Nineteen participants (12.34%) reported having used Couchsurfing before and 75 participants (48.70%) indicated that they would consider using Couchsurfing in the future.

**Figure 3:**
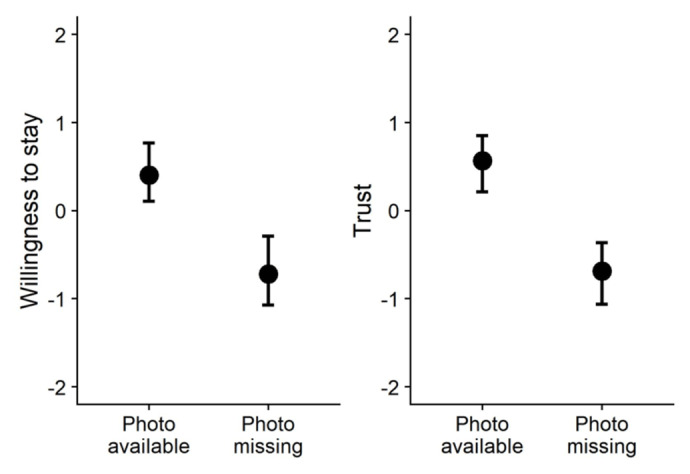
Participants’ willingness to stay with hosts (left panel) and trust in hosts (right panel) with and without profile photos (Study 2). *Note*. The graph displays predicted means and 95% CIs.

First, we tested how participants’ willingness to stay with a host was affected by the presence vs. absence of a profile photo by estimating a multilevel regression model with random intercepts and slopes per listing and per participant. Regressing willingness to stay on a dummy variable indicating whether the profile included a photo (0 = photo missing, 1 = photo available) revealed a positive effect, *β* = 1.111, *SE* = 0.122, 95% CI [0.866, 1.325], *t*(33.88) = 9.13, *p* < .001. In other words, participants were more reluctant to stay with hosts without a profile photo (see Figure 3), replicating results of Study 1. We also tested how trust in hosts was affected by the presence vs. absence of a profile photo. Regressing trust on photo availability revealed a positive effect *β* = 1.255, *SE* = 0.135, 95% CI [1.004, 1.523], *t*(32.45) = 9.30, *p* < .001. Participants trusted hosts without a profile photo less (see Figure 3). Thus, we found that participants were less willing to stay with hosts (replicating results of Study 1) and hosts were trusted less when their profile did not include a personal photo.

We also used the *mediation* package in R (Tingley et al. 2014) to test whether the effect of photo availability on willingness to stay was mediated by trust toward the hosts. As the *mediation* package only supports models with one, but not multiple random effects, we participant-centered trust and willingness to stay ratings. We then estimated a series of models with random intercepts and random slopes for the effect of photo availability per listing. First, we regressed willingness to stay on photo availability, which showed a positive effect, *β* = 1.109, *SE* = 0.097, 95% CI [0.921, 1.289], *t*(17.05) = 11.49, *p* < .001. Second, we regressed trust on photo availability, which also showed a positive effect, *β* = 1.255, *SE* = 0.105, 95% CI [1.038, 1.465], *t*(17.12) = 11.95, *p* < .001. Third, we regressed willingness to stay on photo availability and trust, which showed a positive effect of photo availability, *β* = 0.451, *SE* = 0.063, 95% CI [0.325, 0.565], *t*(21.70) = 7.12, *p* < .001, and a positive effect of trust, *β* = 0.525, *SE* = 0.018, 95% CI [0.491, 0.560], *p* < .001. Applying the *mediate* function (Tingley et al. 2014) to the two latter models yielded a significant indirect effect of photo availability on willingness to stay via trust, *β* = 0.663, 95% CI [0.536, 0.790], *p* < .001. These results suggest that participants’ avoidance of hosts who withhold personal photos is partly explained by increased distrust toward these hosts. Conversely, the partial mediation results also suggest that increased distrust toward hosts is not the only mechanism that explains participants’ lower willingness to stay with hosts without a profile photo.

### Study 3

Previous studies have shown that consumers systematically avoid sellers with certain characteristics, such as unattractive sellers (Jaeger et al. 2019) or sellers from racial minorities (Nødtvedt et al. 2021). For these sellers, disclosing their appearance via a profile photo can potentially decrease consumer interest leading to lower earning opportunities. In other words, although displaying a profile photo may have a positive effect on consumer engagement in general, withholding a profile photo may actually pay off for sellers for whom displaying a profile photo would lead to negative consequences.

Based on this reasoning, we tested three related hypotheses in Study 3. First, we attempted to replicate previous studies on attractiveness biases in the sharing economy (Jaeger et al. 2019; Peng et al. 2020). We tested whether people show a higher willingness to stay with attractive-looking (vs. unattractive-looking) hosts. That is, we experimentally manipulated whether listings were paired with photos of individuals who were rated relatively low or high on attractiveness by a separate sample of participants. People might show a preference for attractive-looking hosts because they are seen as more trustworthy (Batres & Shiramizu 2022), or because attractive faces are processed more fluently, which may lead to the experience of positive affect (Reber, Schwarz & Winkielman 2004). Second, we examine whether different sellers face different penalties when choosing to omit a personal photo. We test whether the absence of a profile photo has a more negative effect for hosts who might experience a benefit from revealing their appearance (i.e., attractive hosts), compared to hosts who might experience a cost from revealing their appearance (i.e., unattractive hosts). Third, we test whether unattractive hosts can create more consumer interest by omitting a profile photo from their listing. That is, we investigate whether an unattractive profile photo or the absence of a profile photo leads to a lower consumer engagement.

#### Methods

This study was preregistered (see https://osf.io/y4e93/).

**Participants**. We used Prolific to recruit participants from the United Kingdom who completed the study in return for £1. We recruited participants between the ages of 18 and 30 because we expected this age group to be more acquainted with (and more likely users of) sharing economy platforms such as Couchsurfing. We preregistered to recruit 150 participants. A sample of 177 participants was collected and in line with our preregistered exclusion criteria, data from one participant (0.57%) who always indicated the same willingness to stay with hosts, one participant (0.57%) who indicated poor or basic English proficiency were excluded, and 24 participants who indicated having completed the study on a cell phone were excluded from analysis, leaving a final sample of 150 participants (62.00% female; *M*_age_ = 23.44, *SD*_age_ = 3.58).

**Materials and Procedure**. We selected 24 profiles of 12 male and 12 female hosts that were also used in Study 1. For each profile, we created three versions. In one version, the host’s profile photo was missing, and a stylized silhouette of a face was displayed. In the other two versions, we randomly matched the profile with an attractive or an unattractive facial photograph. The images were taken from the 10k Faces Database, which contains a large set of images sourced from the internet (Bainbridge et al. 2013). The database also contains average attractiveness ratings for each image. The ratings were provided by a sample of MTurk workers. We selected the 12 individuals with the lowest attractiveness score and the 12 individuals with the highest attractiveness score for men (low attractiveness: *M* = 2.26, *SD* = 0.16, high attractiveness: *M* = 3.84, *SD* = 0.16) and women (low attractiveness: *M* = 1.98, *SD* = 0.31, high attractiveness: *M* = 4.54, *SD* = 0.09) and randomly matched them with a profile of the same gender.

Next, we created three stimulus sets. Each set included all 24 profiles. We varied which third of profiles was displayed without a photo, with an attractive photo, or with an unattractive photo. Participants were randomly assigned to one of the three sets. To measure host preferences, participants were again asked to rate their willingness to stay with a given host on a scale from –3 (*I would not consider staying with this person*) to 3 (*I would really like to stay with this person*). We also measured whether participants had ever made use of Couchsurfing and, if not, whether they would consider doing so in the future.

#### Results

On average, participants’ willingness to stay with the hosts was slightly below the midpoint of our scale (*M* = –0.37, *SD* = 1.88). Participants’ average willingness to stay across all hosts ranged from –2.92 to 2.46 (on our scale that ranged from –3 to 3). The average minimum willingness to stay with specific hosts was –2.73, whereas the average maximum willingness to stay was 2.37. Thus, even though the mean willingness to stay with hosts across all trials and participants was around the midpoint of our scale, there was substantial variation across different hosts and across different participants. Only two participants (1.33%) reported having used Couchsurfing before, but 74 participants (49.33%) indicated that they would consider using Couchsurfing in the future.

First, as in Studies 1 and 2, we tested how participants’ willingness to stay with a host was affected by the presence vs. absence of a profile photo. We estimated a multilevel regression model with random intercepts and slopes per listing and per participant. Regressing willingness to stay on a dummy variable indicating whether the profile included a photo (0 = photo missing, 1 = photo available) revealed a positive effect, *β* = 0.512, *SE* = 0.085, 95% CI [0.356, 0.665], *t*(38.26) = 6.02, *p* < .001. Participants were more reluctant to stay with hosts without a profile photo.

**Figure 4:**
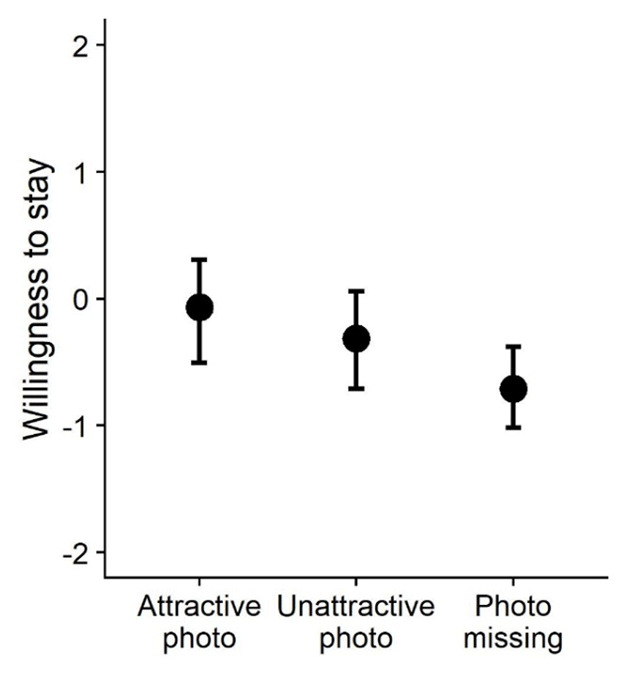
Participants’ willingness to stay with hosts with an attractive, unattractive, or missing profile photo (Study 3). *Note*. The graph displays predicted means and 95% CIs.

Second, we examined the influence of hosts’ attractiveness. Comparing participants’ willingness to stay in the attractive and unattractive host conditions showed the predicted difference, *β* = 0.270, *SE* = 0.060, 95% CI [0.151, 0.389], *t*(123.5) = 4.54, *p* < .001 (see Figure 4). Thus, results showed that participants preferred to stay with attractive over unattractive hosts.

Third, we tested our hypothesis that displaying a profile photo will be more beneficial for attractive (vs. unattractive hosts). Participants indicated a higher willingness to stay with attractive hosts who displayed, rather than withheld, a photo, *β* = 0.648, *SE* = 0.086, 95% CI [0.471, 0.819], *t*(35.71) = 7.51, *p* < .001 (see Figure 4). We also found that participants indicated a higher willingness to stay with unattractive hosts who displayed, rather than withheld, a photo, *β* = 0.378, *SE* = 0.096, 95% CI [0.174, 0.589], *t*(41.73) = 3.95, *p* < .001 (see Figure 4). The positive effect of displaying a photo was significantly larger for attractive hosts, *z* = 2.09, *p* = .036. Thus, we found that showing (vs. not showing) a photo was more beneficial for attractive-looking hosts. However, even for hosts with an unattractive appearance, it was still more beneficial to show, rather than withhold a profile photo.

#### Discussion

In Study 3, we replicated the finding that participants are less willing to stay with hosts who do not include a personal photo in their profile with a sample of participants from the United Kingdom recruited via Prolific. In line with previous work (e.g., Jaeger et al. 2019), we found that participants also showed a preference for attractive-looking hosts. Participants indicated a higher willingness to stay in a home when the profile photo showed an attractive (vs. unattractive host). Crucially, participants were more willing to stay with unattractive hosts than with hosts without a profile photo. Even for hosts with characteristics that are disfavored by consumers (here, an unattractive facial appearance), it was still beneficial to display a personal photo that gives away their appearance, rather than withhold it. In other words, we find that at least with the present study design and photo stimuli, the no-photo penalty was larger than the unattractiveness penalty.

### Study 4

Results of Studies 1–3 suggest that consumers tend to avoid and distrust sellers who choose not to display a profile photo. In Study 4, our primary goal was to test if this preference for profile photos can also be observed in a real-life market. Following previous work on consumer preferences in the sharing economy (e.g., Edelman & Luca 2014), we relied on hedonic price analysis. The core idea behind this approach is that if a good or service has characteristics that are favored by consumers, then this should lead to increased demand (i.e. a higher willingness to pay), which allows sellers to charge higher prices (e.g., Malpezzi 2008).^2^ Conversely, this means that we can understand consumer preferences by examining the association between the price of a good or service and its characteristics.

We leveraged this method to test if consumers prefer sellers who display (rather than withhold) profile photos. We analyzed data from BlaBlaCar (www.blablacar.com), a popular ride-sharing platform that operates in 21 countries, collecting a sample of 461 advertised rides between various cities in Germany. We recorded the price of the advertised rides, whether the driver chose to display a profile photo, and various other attributes that may influence booking decisions (e.g., the driver’s review score, the level of comfort of their car). If consumers prefer sellers with a profile photo, then we should observe a positive association between profile photo presence and prices, controlling for all other relevant attributes of the advertised ride.

We also examined how consumers’ perceptions of sellers based on their profile photo affected their choices. Previous work suggests that consumers favor attractive-looking (Jaeger et al. 2019) or trustworthy-looking hosts (Ert, Fleischer & Magen 2016). We recruited participants to rate drivers’ profile photos to test if attractive-looking, trustworthy-looking, or competent-looking drivers charge higher prices for otherwise similar ratings.

#### Methods

This study was preregistered (see https://osf.io/y4e93/).

**BlaBlaCar Data**. Data on all rides advertised on BlaBlaCar for a pre-defined set of inter-city connections that were offered on three different dates (Sunday, 5 March 2017; Wednesday, 8 March 2017; Friday, 10 March 2017) were collected. Twelve trips across Germany with varying lengths were sampled. The length of the trips ranged from 82 km (Augsburg to Ulm) to 586 km (Berlin to Munich). Data were collected one to three days prior to the ride. Only drivers who had offered at least five rides before and who had received at least one review were selected. For each ride, we recorded the price of the ride and whether a photo of the driver is available. Profile photos were downloaded when available. The profile photos were similar to the types of photos that are displayed on social media platforms. All photos showed the face, and most also showed parts of the upper body of the driver. No other people were displayed in the foreground (one driver was holding a cat). The gender, age, review score (ranging from one to five), driving style (ranging from one to three), and experience of the driver (four levels), the number of offered rides, the number of reviews, whether the trip departed at night (between 10 pm and 6 am), and the comfort of the car (four levels) were also recorded. In total, data on 582 rides were collected.

Rides by drivers who had a high number of offered rides (i.e., 2 standard deviations above the mean) were eliminated and only one ride per driver was included. One ride was removed because the profile photo showed two individuals. Fifty-nine rides were eliminated because data on the driver’s age, experience level, or the car’s comfort level were missing, leaving a final sample of 461 rides.

**Participants**. We recruited 716 US workers from Amazon Mechanical Turk to rate the drivers’ profile photos in exchange for $0.50. Data from 8 participants (1.12%) who always indicated the same rating were discarded, leaving a final sample of 708 participants (*M*_age_ = 34.91, *SD*_age_ = 11.21; 40.5% female).

**Procedure**. Participants saw a random subset of 56 profile photos and were asked to rate the person on trustworthiness, competence, or attractiveness on a 9-point scale ranging from 1 (*not at all* [trait]) to 9 (*extremely* [trait]). Each participant rated the photos on only one trait dimension and each photo was rated by at least 24 participants. The average rating on each trait was calculated as an indicator of perceived trustworthiness, competence, and attractiveness.

#### Results

Multilevel regression models with price as the outcome variable and random intercepts per trip were estimated. The price of the ride, the number of reviews, and the number of rides offered were log_10_-transformed due to their skewed distributions. The driver’s age and the three trait scores were *z*-standardized. We preregistered to control for driving style in our models, but a substantial number of drivers (23.64%) had missing data for this variable. Therefore, we first present analyses that omit driving style as a covariate but are therefore based on a larger number of observations. As a robustness check, we also present analyses that include driving style as a covariate.

Prices of the advertised rides ranged from $4.72 to $57.82 (*M* = $19.97, *SD* = $10.33, *Mdn* = $16.52).^3^ Of the 461 drivers, 400 (84.75%) had a photo available, whereas 61 (15.25%) did not. Drivers were on average 34 years old (*SD* = 10.31) and 83.5% were male. Detailed descriptive statistics are reported in the Supplemental Materials.

First, we tested whether drivers with a profile photo charged higher prices for their rides than drivers without a profile photo (see Table 1, Model 1). In line with this hypothesis, photo availability positively predicted price, *b* = 0.037, *SE =* 0.013, 95% CI [0.013, 0.063], *t*(436.1) = 2.85, *p* = .005. Specifically, drivers with a profile photo charged 8.96% higher prices.

**Table 1:** Associations between the price of advertised BlaBlaCar rides and profile photo availability and the perceived trustworthiness, competence, and attractiveness of the driver.


	MODEL 1	MODEL 2	MODEL 3	MODEL 4

Photo available	0.037**		0.046***	

Trustworthiness		0.0003		0.0004

Competence		0.006		0.004

Attractiveness		–0.006		–0.004

Female	0.010	0.008	0.005	0.003

Age	0.002	–0.002	0.003	0.001

Experience: Experienced	–0.013	–0.016	0.004	–0.004

Experience: Expert	–0.022	–0.022	–0.003	–0.005

Experience: Ambassador	–0.034^†^	–0.038^†^	–0.021	–0.022

Number of rides	0.018	0.015	0.017	0.012

Review score	–0.007	–0.011	–0.035	–0.024

Number of reviews	0.013	0.019	0.021	0.020

Departs at night	–0.031^†^	–0.034^†^	–0.046	–0.046^†^

Comfort of car: 2	–0.034	–0.021	–0.009	–0.009^†^

Comfort of car: 3	–0.019	–0.012	0.005	–0.002

Comfort of car: 4	0.004	0.011	0.028	0.023

Driving style			–0.055	–0.013

Intercept	1.232***	1.277***	1.480***	1.359***

Observations	461	400	352	306


*** *p* < .001, ** *p* < .01, * *p* < .05, ^†^ *p* < .10.

Next, we tested whether drivers that are perceived to be more trustworthy, competent, or attractive charged higher prices for their rides (see Table 1, Model 2). Results showed no association between the price of advertised rides and drivers’ perceived trustworthiness, *b* = 0.0003, *SE =* 0.005, 95% CI [–0.010, 0.010], *t*(373.1) = 0.05, *p* = .96, competence, *b* = 0.006, *SE* = 0.004, 95% CI [–0.003, 0.015], *t*(373.1) = 1.34, *p* = .18, or attractiveness, *b* = –0.006, *SE* = 0.005, 95% CI [–0.015, 0.004], *t*(373.1) = 1.20, *p* = .23.

To test the robustness of our findings, we estimated additional models that included driving style as a covariate but were based on a smaller number of observations. Photo availability still significantly predicted price, *b* = 0.046, *SE* = 0.019, 95% CI [0.004, 0.083], *t*(326.2) = 2.37, *p* = .018, (see Table 1, Model 3), which translated to an 11.20% price premium for drivers with a photo. We again observed no association between price and drivers’ perceived trustworthiness, *b* = 0.0004, *SE* = 0.006, 95% CI [–0.010, 0.011], *t*(280.2) = 0.08, *p* = .94, competence, *b* = 0.004, *SE* = 0.005, 95% CI [–0.007, 0.014], *t*(280.1) = 0.70 *p* = .48, or attractiveness, *b* = –0.004, *SE* = 0.006, 95% CI [–0.016, 0.007], *t*(280.1) = 0.73, *p* = .47 (see Table 1, Model 4).

#### Discussion

In line with our experimental data, the results of our hedonic price analysis suggest that consumers prefer sellers with profile photos. BlaBlaCar drivers without a profile photo charged approximately 9–11% lower prices compared to drivers with a profile photo. This disparity emerged after controlling for a host of other characteristics that could explain the photo-related price difference, including drivers’ review score, experience, gender, and age.

## General Discussion

Sharing economy sellers may choose not to display a profile photo because of general privacy concerns (Dillahunt & Malone 2015; Penman, Vedantam & Nesterak 2016; Ranzini et al. 2017) or because they wish to conceal their appearance or social identity to prevent discrimination or other unfavorable treatment by consumers (Jaeger et al. 2019; Nødtvedt et al. 2021; Troncoso & Luo 2023). Here, we tested whether this strategy comes at the cost of decreased consumer trust and engagement. Trust is an important predictor of consumer decision-making in the sharing economy and consumers rely on profile photos (next to other cues) to assess the trustworthiness of sellers (Ert & Fleischer 2019; Kong et al. 2020). Thus, they may avoid sellers without a profile photo. Four preregistered studies supported this hypothesis, showing that consumers distrust and avoid sellers without a profile photo.

In Studies 1–3, we showed participants simplified but realistic Couchsurfing profiles and measured their (hypothetical) willingness to stay with different hosts. Crucially, we manipulated whether hosts displayed a personal photo on their profile. We found consistent evidence that, all other aspects of the profile being equal, participants were less willing to stay with hosts when they did not display a profile photo. Specifically, analyzing the aggregated data from all three studies (see Supplemental Materials), participants’ willingness to stay with a host was 0.8 points lower on a 7-point scale when the host was not displaying a personal photo. These findings are in line with previous work by Fagerstrøm and colleagues (2017), who found similar results for consumer preferences for Airbnb hosts. In their study, participants’ preferences for profile photos were perhaps less surprising given that the photos were the only piece of personal information about hosts. Our results show that participants penalize hosts without a profile photo even when they have access to other personal information about them (i.e., gender, age, and an ‘about me’ section). We explore the role of some of these factors in the Supplemental Materials (see Figure S1). In short, aggregating data from Studies 1–3, we found that female hosts were preferred over male hosts, but this was only observed for female participants. We did not find an effect of hosts’ apparent ethnic background, but this may have been because our sample did not contain many non-White hosts.

Our studies also extend previous work on the topic. In line with the idea that profile photos reduce anonymity and increase consumer trust, we found that participants reported lower levels of trust toward hosts when they did not display a profile photo and participants’ trust toward hosts was positively related to their willingness to stay with them (Study 2). We also found that trust partially mediated the effect photo availability on participants’ willingness to stay with hosts. These results suggest that, although trust likely plays an important role, there are also other psychological mechanisms that explain participants’ lower willingness to stay with hosts without a profile photo. More work is needed to explore these mechanisms. For example, it is possible that the ease with which judgments can be formed in the presence of a facial image, due to the fluent processing of faces (Freeman & Johnson 2016; see also Reber et al. 2004), may lead to positive affect and, ultimately, more positive assessments of the host (Winkielman & Cacioppo 2001).

Results of Study 3 shed more light on the size of the no-photo penalty. In line with previous work (e.g., Jaeger et al. 2019; Peng et al. 2020), we found that participants favored attractive-looking over unattractive-looking Couchsurfing hosts. This may indicate that at least some hosts could be better off not sharing a profile photo if it discloses an appearance or group membership that leads to consumer disengagement. Yet, in our study, even hosts with an unattractive appearance were more popular when they displayed (vs. withheld) a profile photo. Participants were more willing to stay with unattractive hosts than with hosts without a profile photo. Thus, we found that the no-photo penalty was larger than the unattractiveness penalty.

Finally, going beyond preferences in hypothetical sharing economy interactions, Study 4 tested if consumers’ preference for profile photos can also be observed in a real-life market. A hedonic price analysis (Malpezzi 2008; Rosen 1974) of 461 rides advertised on BlaBlaCar, a long-distance ride-sharing market, revealed that drivers with a profile photo charged approximately 9–11% higher prices for otherwise similar rides (i.e., after controlling for a host of other factors that could vary between drivers who display vs. omit profile photos). This price premium suggests that consumers prefer sellers with profile photos, which allows them to charge higher prices. In contrast to previous work that examined data from Airbnb (Ert, Fleischer & Magen 2016; Jaeger et al. 2019), we did not find evidence suggesting that consumers favor attractive-looking or trustworthy-looking BlaBlaCar drivers.

In sum, our studies provide converging evidence that people prefer sharing economy sellers who display a personal photo. Although our studies were not designed to compare the effect of different types of information on consumer preferences (and we do not claim that photo availability is necessarily more important than other cues), the present results suggest that the effect of photos is not negligible. Our experimental results showed a relatively large effect of displaying a profile photo (a 0.8-point increase on a 7-point scale; see Supplemental Materials). Moreover, the estimated price premium of 10% for displaying profile photo on BlablaCar (Study 4) was similar to, for example, the effect of hosts’ race on the prices of Airbnb listings (10% difference for Black vs. White hosts; Jaeger et al. 2019). For comparison, the effect observed here was larger than the effect of a one-standard-deviation increase in review score (ca. 5%), but smaller than the effect of an additional bedroom (ca. 16%; Jaeger et al. 2019).

### Theoretical and practical implications

The present results are in line with more general theoretical models of consumer decision-making in the sharing economy, which highlight that consumers (a) assess the trustworthiness (or other relevant characteristics) of sellers based on information that is provided by the platform, and (b) rely on these assessments when choosing between sellers (e.g., Ert, Fleischer & Magen 2016). In line with previous work (Ert & Fleischer 2019; Mayayise 2023; Teubner et al. 2014), results of Study 2 suggest that consumers rely on personal photos of Couchsurfing hosts to evaluate their trustworthiness. In turn, trust toward a host predicted booking intentions in Study 2 (Ert, Fleischer & Magen 2016; Kong et al. 2020; Teubner et al. 2014). Our analysis of preferences for BlaBlaCar drivers were more mixed. Although we found support for our primary hypothesis that drivers who share a personal photo can charge higher prices for similar ride offers, we did not find that the perceived trustworthiness of drivers was associated with asking prices (null results were also found for perceptions of competence and attractiveness). Getting into the car of a stranger should present a significant perceived security risk for many potential customers, which led us to expect that trustworthiness perceptions of drivers should be an important determinant of consumer preference. These null results are surprising, and we can only speculate about their cause. It is possible that other characteristics are more important when selecting rides on this platform, such as perceived driving ability, which we did not measure in our study.^4^ It is also possible that our sample of 400 drivers was not large enough to detect smaller effects of perceived trustworthiness. More work is needed to explore these possibilities.

Our results also highlight an important part of sellers’ ‘privacy calculus’ (Dinev & Hart 2006; Olivero & Lunt 2004). Sellers are thought to weigh the potential costs and benefits when deciding which information to disclose. In the present case, we find that the benefits of increased anonymity from not posting a profile photo can have significant costs in the form of lower consumer trust and engagement, which can ultimately lower sellers’ expected earnings. Thus, sellers must engage in tradeoffs because of a direct conflict between anonymity and economic benefits. Sellers should also be aware that when weighing privacy concerns against higher expected earnings, the priorities of sellers and companies may not be aligned. Although both individual sellers and sharing economy platforms profit with higher consumer engagement, only sellers bear the costs of sharing personal information (and potentially becoming the target of discrimination).

### Limitations and future directions

We used two approaches with complementary strengths and limitations to investigate whether consumers avoid sellers without a profile photo. In Study 4, we analyzed real-word data from BlaBlaCar. Preferences for drivers with (vs. without) a photo should lead to stronger demand for their rides, which would allow them to charge higher process for otherwise equivalent rides. To isolate the influence of profile photos, we controlled for a host of other attributes that may vary between drivers who show vs. withhold a photo and which may influence consumer preferences. Still, we can only draw limited inferences about the causal role of profile photos on consumer choices based on this correlational design. There may be other cues (a) that are correlated with whether drivers share a personal photo and (b) that people rely on when choosing drivers (such as the driver’s perceived ethnic background; Farajallah et al. 2016; Ge et al. 2020).

Stronger evidence for the causal role of profile photos was provided by the results of Studies 1–3, in which we measured hypothetical preferences for Couchsurfing hosts and manipulated the availability of a profile photo while keeping all other listing information constant. Thus, although our study designs varied in how closely they approximate real life decision processes in the sharing economy and how strongly we can infer a causal relation between photo availability and consumer choice from the results, converging results from all four studies provide stronger evidence for such an effect.

Although participants in our studies had access to more information about hosts than in previous studies (which should provide a more accurate estimate of the impact of profile photos on choices), we did remove hosts’ name to provide some level of anonymity. If people are more likely to trust hosts who disclose their name, then removing this cue may also inflate the importance of (and participants’ reliance on) profile photos. This limitation did not apply to the results of Study 4, where we examined real-world price data for listings that contained the driver’s name, photo, and other personal information. The present results provide more robust evidence that profile photos affect consumer choice—even when people have access to other (personal) information about sellers—but more research is needed to better understand how much weight people put on photos, names, and other cues to a seller’s identity and trustworthiness.

The primary goal of the current studies was to explore how consumers react to the presence versus absence of profile photos. Our studies suggest that not sharing a personal photo on one’s profile may come at a cost (in the form of lower demand), but more work is needed to understand whether sharing economy sellers are aware of this potential cost. Anecdotal evidence suggests that Airbnb hosts from racial minorities withhold (or display fake) profile photos to hide their ethnic background because of experiences with discrimination (Penman, Vedantam & Nesterak 2016). One study with mostly African American sharing economy participants found that the majority of participants reported a reluctance to disclose photos and other personal information due to privacy and security concerns. However, the sample size of this study was very small (*n* = 20). Additional studies are needed to systematically understand which information sellers are most reluctant to share, why sellers may choose to withhold a profile photo, whether some sellers experience stronger concerns, and whether this influences their participation and experiences in the sharing economy.

Several factors could constrain the generalizability of our findings. Our studies focused on sharing economy interactions, during which consumers can expect to have direct contact with the seller. It is plausible that many consumers perceive it as somewhat risky to enter the home (in the case of Couchsurfing) or car (in the case of BlaBlaCar) of a complete stranger. Assessing sellers’ trustworthiness, for example, on the basis of profile photos is of particular important under these circumstances. It is possible that we would observe different results for sharing economy interactions that involve less or no personal contact on platforms where cues other than the sellers’ profile photo are displayed very prominently. For example, when renting an entire apartment via Airbnb, it is common that people only have brief contact with a host outside of the rental to pick up the keys (or no contact at all of the host is using a lock box).

While profile photos may have a weaker effect on consumer preferences in some situations, previous research suggests that photos still matter to some extent under these circumstances. Studies from different countries have consistently shown that people prefer Airbnb hosts from the ethnic majority (Fagerstrøm et al. 2017; Marchenko 2019), but evidence on whether these effects are stronger when the apartment is shared with the host (i.e., when there is more contact) are mixed (Jaeger & Sleegers 2023). Effects of sellers’ race and attractiveness have also been found for platforms that involve no personal contact, such as eBay (Doleac & Stein 2013) or a peer-to-peer money lending platform (Ravina 2008). These findings are perhaps not surprising, given that assessing a seller’s trustworthiness is not only relevant for personal safety (which applies to the sharing economy transactions we focused on), but also for confidence in the seller’s motivation to provide the product or service as it is advertised (which applies to the sharing economy more broadly). Still, whether similar effects would be observed for the presence of profile photos on these platforms remains an open question.

Results of Studies 1–3 were based on samples of relatively young participants from Western European countries. We do not consider participants’ age to be a limitation of the current studies (in fact, we explicitly sampled young participants from Prolific in Study 3) because sharing economy platforms such as Couchsurfing, BlaBlaCar, and Airbnb are primarily used by young adults. Although a minority of participants in our sample reported having used Couchsurfing themselves, around 50% indicated that they would be open to using it in the future. Additional work is needed to test if the present results replicate with participants from other cultures, especially in non-Western samples. An analysis of Airbnb profiles in China and the United States found that Chinese hosts were less likely to display a personal photo (Jang 2023). Moreover, although hosts without a photo received lower review scores in both countries, the difference was larger in the United States. Studies that examined racial price disparities on Airbnb across different markets also found considerable cross-country variation (Jaeger & Sleegers 2023). More work is needed to map and explain these differences.

Future work could also explore if the presence of other information can compensate for the negative effect that the absence of a personal photo has on consumer preferences. For example, it is possible that consumers tolerate the absence of a profile photo if there are diagnostic cues that suggest that a seller can be trusted, such as a very high review score or an indication that the personal identity of a seller has been verified and recorded by a platform. Relevant results from previous studies were mixed. On the one hand, Nødtvedt and colleagues (2021) found that racial bias in participants’ hypothetical choices of Airbnb listings disappeared for listings that received the highest possible review scores (compared to apartments that received a mediocre score). On the other hand, Fagerstrøm and colleagues (2017) found that participants still avoided Airbnb listings with missing profile photos when the listing had the highest possible review score. Review scores may seem like the most relevant and relatively objective indicator that consumers could rely on instead of profile photos and other personal information. However, on many sharing economy platforms, review scores may not be diagnostic as they are uniformly high with little variation undermining their informativeness (e.g., Marchenko 2019).

## Data Accessibility Statement

All data, analysis scripts, and preregistration documents are available at the Open Science Framework (https://osf.io/y4e93/). As advised by our data protection officer, we do not share the stimuli used in our studies to protect the privacy of the hosts and drivers. We report how our sample sizes were determined, all data exclusions, and all measures in the studies. We highlight instances in which we diverged from the preregistered plan.

## Footnotes

We do not conceive of distrust as a separate construct but use the term to denote low(er) levels of trust. Some research in this area focuses on trustworthiness judgments, instead of trust, but these terms share considerable conceptual overlap, as trustworthiness judgments reflect a key part of the definition of trust (‘positive expectations of the intentions or behavior of another’).

Sellers could also increase supply to meet demand. However, in many cases, supply is relatively inflexible. Airbnb hosts who rent out an extra room in their apartment cannot increase their supply even when their listing is extremely popular because they can only rent out their room to one occupant on any given night. They can, however, easily increase the price of their listing to capture demand. In sharing economy markets, supply is relatively inflexible, which means that prices should be more indicative of demand.

Original prices were in Euro. Prices were converted to US dollars using an exchange rate of 1.18 (www.x-rates.com, accessed on 2 August 2017).

One might expect that competence perceptions may reflect perceived driving ability in a ride sharing context, but the participants who rated drivers’ trustworthiness, competence, and attractiveness did not know that the photos were taken from BlaBlaCar.
